# Short Report: Intervention of Reading and Spelling Problems in Children With Co‐Occurring Attention‐Deficit Hyperactivity Disorder and Dyslexia

**DOI:** 10.1002/dys.70032

**Published:** 2026-04-01

**Authors:** Cara T. Verwimp, Nelina Meiboom, Jurgen Tijms

**Affiliations:** ^1^ Research Institute of Child Development and Education University of Amsterdam Amsterdam the Netherlands; ^2^ Rudolf Berlin Center University of Amsterdam Amsterdam the Netherlands; ^3^ Samenwerkingsverband Driegang Gorinchem the Netherlands; ^4^ Youz, Center for Youth Mental Healthcare Rotterdam the Netherlands; ^5^ RID Center for Learning Disabilities Amsterdam the Netherlands

**Keywords:** ADHD, co‐occurrence, dyslexia, intervention

## Abstract

Despite the high co‐occurrence of dyslexia and attention‐deficit hyperactivity disorder (ADHD), dyslexia is undertreated in children with ADHD. In this study, we aimed to investigate whether standard intervention for dyslexia is as effective for children with co‐occurring ADHD and dyslexia as for those with dyslexia only. Children with co‐occurring ADHD and dyslexia (*n* = 16), and with dyslexia only (*n* = 16) received a phonics‐based intervention for dyslexia. Reading and spelling skills were tested at the start and at the end of intervention. Bayesian repeated‐measures ANOVAs revealed that children in the co‐occurring ADHD and dyslexia group had, on average, poorer levels of reading and spelling proficiency at the baseline. Intervention was equally effective in children with ADHD and dyslexia as it was in those with dyslexia only for reading fluency. For spelling, our results suggested stronger gains in the ADHD and dyslexia group than in the dyslexia only group. These results suggest that the effectiveness of intervention for dyslexia was not negatively affected by the presence of co‐occurring ADHD.

## Practitioner Points

- Children with co‐occurring ADHD and dyslexia benefit from dyslexia intervention in their reading and spelling skills.
- Responsiveness to dyslexia intervention is not reduced in children with co‐occurring ADHD.
- Including dyslexia intervention in protocols for children with ADHD and dyslexia is recommended.

## 1 Introduction

Service delivery systems are typically organised in professional and diagnostic silos, where children are treated for their primary disorder, often without systematic assessment and targeted intervention for potential co‐occurring problems (Boulton et al. 2021; Leve 2016). Accordingly, intervention protocols are based on evidence mostly derived from single disorder samples in which comorbidities are excluded despite the high co‐occurrence of developmental disorders (Astle et al. 2022; Bearman and Weisz 2015; Dewey 2018).

Recent conceptualizations of developmental disorders challenge this categorical approach, viewing them as a family of associated disorders rather than discrete categories, with multiple risk and protective factors spanning diagnostic boundaries. This transdiagnostic perspective proposes that intervention should be guided by symptoms and atypical neurocognitive mechanisms, irrespective of the classified diagnostic category (Astle et al. 2022; Cuthbert 2022; Dewey 2018). However, adopting such integrated intervention approaches requires an important intermediate step: establishing whether interventions validated in single‐disorder samples maintain their effectiveness when diagnostic boundaries are crossed. Without evidence that interventions generalise to the comorbid presentations commonly seen in clinical practise, clinicians lack the empirical basis needed to move beyond diagnostic silos. This knowledge gap is particularly problematic for frequently co‐occurring conditions like attention‐deficit hyperactivity disorder (ADHD) and developmental dyslexia.

About 25%–40% of children with ADHD also meet the diagnostic criteria for dyslexia (Drechsler et al. 2020; Maughan and Carroll 2006; Willcutt et al. 2012). Moreover, longitudinal studies indicate that children with co‐occurring ADHD and dyslexia have a significantly higher risk of poor academic attainment, low occupational status in adulthood, and socio‐emotional problems than children with ADHD only (Boada et al. 2012; von Wirth et al. 2022). Although poor academic performance is one of the most prominent reasons for clinical referral, intervention plans for children with ADHD often lack targeted intervention for co‐occurring reading and spelling problems. Standard intervention approaches for children with ADHD typically include pharmacological and behavioural interventions, and primarily focus on attentional and behavioural symptoms (Caye et al. 2019; Pliszka 2007). However, these interventions fail to have an additional impact on these children's reading or spelling problems (Kortekaas‐Rijlaarsdam et al. 2019; Tannock et al. 2018). One potential reason might be that the comorbid condition is associated with specific neurocognitive dysfunctions (e.g., in the domain of phonological‐orthographic processing) that are not addressed by either the pharmacological or the nonpharmacological ADHD intervention (Froehlich et al. 2018). At the same time, clinical reports suggest that intervention for the primary ADHD symptoms might be less effective in children with a co‐occurring reading disorder (Bramble 2007), suggesting that the persisting reading problems and associated frustrations, and negative self‐concept might hinder the improvement of behavioural or attentional symptoms within the school context.

Intervention of dyslexia is a well‐studied subject, and intervention studies have supported its effectiveness (Galuschka et al. 2014). These findings refer to children with dyslexia only, as children with co‐occurring disorders are commonly excluded. An exception is a study of Tannock et al. (2018), that showed significant increases in reading skill in children with co‐occurring ADHD and dyslexia following a dyslexia intervention. It, however, remains unclear if and to what extent the presence of co‐existing ADHD affects children's responsiveness to dyslexia intervention. It is well known that the effectiveness of a recommended intervention for the primary condition can be moderated by the presence of co‐occurring conditions (Dewey 2018; Thapar et al. 2017). To support the use of intervention of reading disabilities in children with co‐occurring ADHD and dyslexia, it is important to gain insight to what extent the body of evidence for the effectiveness of dyslexia intervention is generalisable to children with co‐occurring ADHD and dyslexia. The objective of the present study was therefore to compare the effects of dyslexia intervention for children with co‐occurring ADHD and dyslexia to those of children with dyslexia only.

## 2 Method

### 2.1 Participants

All children were referred to a clinic for child and adolescent psychiatry in the Netherlands, that also has a special department for children with learning disabilities. The dyslexia‐only group included children who entered the learning disabilities department at the same recruitment period as those with ADHD+dyslexia and were matched on age. Presence of dyslexia, ADHD and optional other diagnoses was based on receiving a formal diagnosis at the clinic. For ADHD diagnosis, children received an extensive diagnostic procedure by psychologists and psychiatrists. A DSM‐5‐based clinical diagnosis was established based on data from multiple sources (interviews; observations; psychological assessment; psychiatric examination). Diagnosis of dyslexia was also based on an extensive diagnostic assessment, by a psychologist, and in line with DSM‐5, using data from multiple sources (interviews, school records, psychological assessment). To be eligible, participants had to show a persistent, specific reading deficit, that is, persistently scoring a percentile score of 10 or lower on standard reading fluency measures at three consecutive timepoints during a period of at least one school‐year, whilst having received weekly remedial teaching at school for at least half a school‐year, and an IQ‐score greater than 80.

Our sample included 16 children with ADHD+dyslexia (age (in months): M = 109.75; SD = 14.49; 25% female), and 16 children with dyslexia only (age (in months): M = 108.81; SD = 13.67; 50% female). A chi‐squared test showed no significant difference in female–male ratio between the two groups (*Χ* ^2^ (1) = 2.13, *p* = 0.14). Additionally, Bayesian *t*‐tests revealed no differences between the two groups in intelligence (BF_10_ = 1.30), vocabulary (BF_10_ = 0.35), and socioeconomic status (BF_10_ = 0.35; for details see Supporting Information 1). In the ADHD+dyslexia sample, six of the children met the criteria of a third diagnosis in addition to ADHD and dyslexia (five autism spectrum disorder (ASD), one post‐traumatic stress disorder (PTSD)). Note that these numbers are in line with co‐occurrence rates reported in the literature (Drechsler et al. 2020). Analyses showed no significant differences in pre‐test or post‐test scores between those with and without a third diagnosis (for details see Supporting Information 2), therefore we treated them as one group in the further analyses. Children in the ADHD+dyslexia sample received standard care in relation to ADHD, that is, three sessions of psychoeducation on ADHD for the children and their parents, and in most cases methylphenidate medication. This study obtained ethical approval from the ethics committee of the University of Amsterdam. Written consent was obtained from the parents.

### 2.2 Procedure and Intervention

Participants received a baseline assessment (pre‐test), followed by the intervention programme, and a post‐test was conducted after finishing the intervention. The intervention followed the standard dyslexia intervention protocol (Galuschka et al. 2014) and included structured, systematic phonics instruction. Children received weekly, 50‐min one‐on‐one sessions at the clinical centre. Intervention was provided by (child or educational) psychologists and consisted of approximately 40 weekly sessions. As many children with dyslexia have poor spelling skills in addition to their reading disability, the intervention addressed both reading and spelling skills. See Fraga González et al. (2015) for a more elaborate description of the intervention method. Assessments were conducted by clinicians at the clinic.

### 2.3 Outcomes

Word reading fluency was measured by two tests that both measure the number of isolated words read correctly within a limited time, that is, 3DM Reading Test (3DM; reliability: *r* = 0.95, test–retest (Blomert and Vaessen 2009)), and One‐Minute‐Test (OMT; reliability: *r* = 0.89 to 0.93, test–retest (Brus and Voeten 1999)). Spelling accuracy was measured by a computerised test that required the child to choose the correct spelling of a word out of four alternatives (3DM Spelling Test; reliability: *r* = 0.80, internal consistency (Blomert and Vaessen 2009)).

### 2.4 Statistical Analysis

As we wanted to test whether intervention effects in children with co‐occurring ADHD and dyslexia were comparable to those of children with unimorbid dyslexia, as opposed to less effective, we opted for a Bayesian analytic approach. Bayesian approaches have the advantage of estimating evidence for both the null and alternative hypothesis, whereas frequentist methods only provide evidence against the null hypothesis (Dienes 2014). More precisely, we used Bayesian repeated‐measures analyses of variance to compare gains in reading fluency and spelling of children with co‐occurring ADHD and dyslexia to those of children with dyslexia only. We present all models compared against the best fitting model, and report two Bayesian factors, that is, BF_10_ and the BF_Inclusion_. BF_10_ quantifies evidence against the best model. BF_Inclusion_ is based on Bayesian model averaging and indicates the probability that a certain factor has to be included in an analysis of variance relative to the case where it is not. We used Jeffrey's benchmarks to interpret the strength of evidence; BFs between 1 and 3 were considered anecdotal, BFs between 3 and 10 as moderate, between 10 and 30 as strong, between 30 and 100 as very strong and > 100 as decisive evidence for a given model relative to another (Wagenmakers et al. 2018). Analyses were run using JASP statistical software (v0.17). Due to technical problems with a computer, the data for the 3DM test was missing for one data point for one participant of the ADHD+dyslexia group, consequently this participant was excluded in the analyses of 3DM reading fluency and spelling.

## 3 Results

**FIGURE 1:**
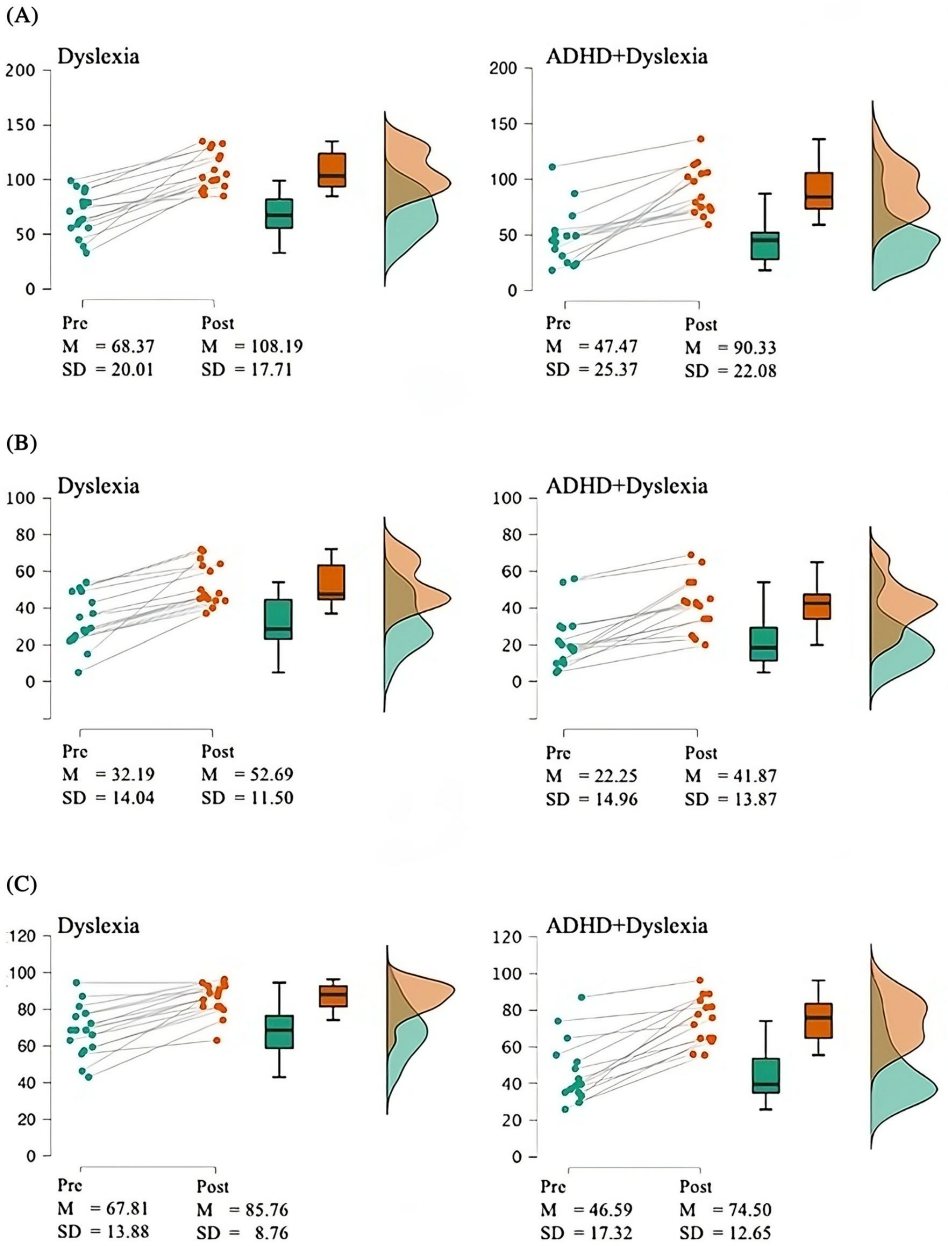
Raincloud plots of pre‐test and post‐test scores per group, including mean and SD, for (A) Word Reading Fluency – 3DM, (B) Word Reading Fluency – OMT, and (C) Spelling – 3DM.

Pre‐test and post‐test scores on both word reading fluency measures are depicted in Figure 1A,B. Using a Bayesian repeated measures ANOVA, we tested for main effects of the factors group and time, as well as for an interaction effect. Results showed that the model including main effects for time and group was the best fitting model for both word reading fluency measures, see Table 1. The model including both main effects was about three times more likely than the model with the additional interaction effect, providing anecdotal to moderate support for the former model. Analysis of the BF_Inclusion_ showed for both reading fluency measures that, averaged across all candidate models, the data strongly support inclusion of main factor time (BF_incl_ = 3.58 × 10^10^ (3DM), 7.80 × 10^8^ (OMT)) as well as group (BF_incl_ = 6.02 (3DM), 2.34 (OMT)), but do not provide substantial support for the interaction between the two factors (BF_incl_ = 1.24 (3DM), 0.99 (OMT); for details see Supporting Information 3). Note that a tendency, if any, towards an interaction effect is in the opposite direction as that of our alternative hypothesis of less effect in the ADHD+dyslexia sample. Thus, results indicate growth in reading proficiency for both groups, and poorer reading levels for the ADHD+dyslexia group, but no evidence for reduced effectiveness in the ADHD+dyslexia sample.

**TABLE 1:** Model comparison for word reading fluency and spelling.

Models	P(M)	P(M|data)	BF_M_	BF_10_	Error %
A. Reading fluency 3DM					
Time + group	0.200	0.664	7.890	1.000	
Time + group + time × group	0.200	0.237	1.241	0.357	2.647
Time	0.200	0.100	0.443	0.150	1.837
Group	0.200	1.227 × 10^−11^	5.110 × 10^−11^	1.925 × 10^−11^	2.132
Null model	0.200	5.867 × 10^−12^	2.347 × 10^−11^	8.841 × 10^−12^	1.626
B. Reading fluency OMT					
Time + group	0.200	0.580	5.527	1.000	
Time	0.200	0.222	1.141	0.383	2.683
Time + group + time × group	0.200	0.198	0.987	0.341	2.993
Group	0.200	5.475 × 10^−10^	2.190 × 10^−9^	9.438 × 10^−10^	2.563
Null model	0.200	3.073 × 10^−10^	1.229 × 10^−9^	5.298 × 10^−10^	2.017
C. Spelling					
Time + group + time × group	0.200	0.638	7.055	1.000	
Time + group	0.200	0.354	2.190	0.554	3.540
Time	0.200	0.008	0.032	0.013	2.864
Group	0.200	1.040 × 10^−9^	4.159 × 10^−9^	1.629 × 10^−9^	2.638
Null model	0.200	8.409 × 10^−11^	3.364 × 10^−10^	1.318 × 10^−10^	2.540

*Note:* All models include subject and random slopes for all repeated measures factors.

Pre‐test and post‐test scores on spelling are depicted in Figure 1C. For spelling, our results revealed that the model including main effects for time and group and the interaction effect was the best fitting model, although the evidence in favour of this model, vis‐à‐vis the model with only main effects of time and group, was only anecdotal. Analysis of the BF_Inclusion_ showed that, averaged across all candidate models, the data strongly support inclusion of both main factors time (BF_incl_ = 5.93 × 10^8^) and group (BF_incl_ = 82.65), as well as provide substantial support for the interaction between the two factors (BF_incl_ = 7.05; for details see Supporting Information 3). As can be seen in Figure 1C, the interaction effect points towards a more pronounced growth in spelling proficiency in the ADHD+dyslexia group, that is, in the opposite direction as that of our alternative hypothesis (less effective in comorbid ADHD+dyslexia sample). Thus, results indicate poorer spelling levels for the ADHD+dyslexia group, and growth in spelling for both groups that appears to be somewhat stronger in the ADHD+dyslexia group.

Finally, to examine whether the children improved their reading and spelling proficiency compared to the general age‐related population, we compared the standard scores (T‐scores, having a general population mean of 50, and SD of 10) on the pre‐test and post‐test for reading (3DM) and spelling. For this analysis, we pooled the data of the comorbid and unimorbid samples. Bayesian paired sample *t*‐tests revealed decisive evidence in favour of the alternative hypothesis, that is, standard scores at post‐test were greater than at pre‐test for both reading (pre: M = 30.62, SD = 6.69, post: M = 38.23, SD = 8.23; BF > 100) and spelling (pre: M = 34.84, SD = 9.91, post: M = 46.29, SD = 10.17; BF > 100). This result indicates that the participants improved their positions, on average for reading from percentile 2.6 to percentile 12.0 and for spelling from percentile 6.5 to percentile 35.5, within the distribution of reading and spelling ability of a large age‐related normative sample.

## 4 Discussion

Despite its high co‐occurrence with ADHD, dyslexia is undertreated in children with ADHD. This appears to be a consequence of a categorical, single disorder perspective in both research and service delivery systems. To contribute to the development of a more integral, transdiagnostic support model, we examined whether standard phonics‐based intervention for dyslexia is as effective for children with co‐occurring ADHD and dyslexia as for those with dyslexia only. We therefore compared the effects of intervention on reading and spelling skills in both groups, using Bayesian repeated‐measures analyses of variance. Our results showed that children in the ADHD+dyslexia group had, on average, poorer levels of reading and spelling proficiency at the baseline. This finding is in line with reports that comorbidity in neurodevelopmental disorders usually involves more severe symptoms than present in unimorbid cases (Boada et al. 2012; Dewey 2018). In this specific case, as the attention network is known to be involved in reading acquisition (Chyl et al. 2021; Verwimp et al. 2023), the attentional problems of children with co‐occurring ADHD and dyslexia might have had an additive, negative impact on their reading and spelling development.

Concerning our main objective, it was shown that both groups showed improvements in reading fluency, and that the intervention was equally effective in children with ADHD and dyslexia as it was in those with dyslexia only. For spelling, our results suggested stronger gains in the ADHD+dyslexia than in the dyslexia only group. This latter finding might be related to the structured, explicit character of the intervention approach and the one‐to‐one intervention setting, which potentially provides extra support for children with attentional difficulties. In accordance with previous findings (Fraga González et al. 2015; Tijms 2011), we found that following the intervention, the participants improved their positions within the distribution of reading and spelling ability of a large age‐related normative sample.

An important consideration in interpreting these findings is that most children in the ADHD+dyslexia group received standard ADHD care, including methylphenidate medication. Withholding such care would have been neither ethically justifiable nor clinically representative. Importantly, there is substantial evidence that standard ADHD interventions, including stimulant medication, do not remediate reading and spelling difficulties (Kortekaas‐Rijlaarsdam et al. 2019; Tannock et al. 2018). Therefore, it is unlikely that the observed gains in reading and spelling can be attributed to ADHD medication alone. Rather, the present findings indicate that within an integral intervention context, where ADHD symptoms are managed according to standard care, phonics‐based dyslexia intervention remains effective for children with co‐occurring ADHD and dyslexia. This aligns with the primary objective of the study, which was not to isolate intervention components, but to examine whether standard dyslexia intervention retains its effectiveness in a clinically representative comorbid sample.

Taken together, our results revealed that the presence of co‐occurring ADHD did not negatively affect the intervention responsiveness of children with dyslexia. As such, our findings can be interpreted as evidence that the standard dyslexia intervention protocol is also indicated in children with co‐occurring ADHD. In the context of the pertinent research showing that standard interventions for the behavioural and attentional problems in children with ADHD fail to have an impact on co‐occurring reading disabilities in children with ADHD (Barbaresi et al. 2020; Kortekaas‐Rijlaarsdam et al. 2019; Tannock et al. 2018), and that reading disabilities are an important negative predictor of later employment status and other markers of quality of life in children with ADHD (Boada et al. 2012; von Wirth et al. 2022), our results support the recommendation of a combined intervention approach that addresses both behavioural and attentional problems as well as reading problems in children with ADHD and co‐occurring reading disabilities.

More general, this study underpins the recruitment of more ‘natural’ samples that reflect the high incidence of multimorbidity in neurodevelopmental disorders in research studies, instead of the focus on single disorder samples in which comorbidities are excluded. From a clinical perspective, our results support transdiagnostic approaches towards more integrated intervention programmes that match the individuals' needs as well as a more collaborative care system, to obtain improved outcomes for children with neurodevelopmental challenges (Boulton et al. 2021; Holmes et al. 2019).

### 4.1 Limitations

The study used small sample sizes. It is thus important to replicate these findings in larger samples, ideally in a dimensional design in which ADHD‐symptoms are included as covariates of intervention effectiveness, instead of the binary approach of either or not diagnosed. A large‐scale trial might also provide a more in‐depth insight into individual factors mediating intervention responsiveness and thereby inform protocols for more individualised support. As this study relied on clinical data, assessor blinding was not implemented. Although assessors were unaware of which children participated in the study, they were not consistently blinded to the intervention condition. In a future replication study, implementing assessor blinding would be advisable to minimise the risk of bias associated with non‐blinding. Finally, our sample included only children with a formal diagnosis of both ADHD and dyslexia. As such, the findings are specific to this comorbid group and do not generalise to children with ADHD who experience subclinical reading difficulties without meeting full diagnostic criteria for dyslexia. Future research examining the effectiveness of reading interventions across the full spectrum of reading difficulties in children with ADHD would provide a comprehensive clinical insight.

## Funding

This work was supported by the H2020 Marie Skłodowska‐Curie Actions (813546).

## Disclosure

The authors have nothing to report.

## Conflicts of Interest

The authors declare no conflicts of interest.

## Supporting information


Supporting Information: 1.

Supporting Information: 2.

Supporting Information: 3.

## Data Availability

The data that support the findings of this study are available from the corresponding author upon reasonable request.
