# Supplementary material for: Short Report: Intervention of Reading and Spelling Problems in Children With Co‐Occurring Attention‐Deficit Hyperactivity Disorder and Dyslexia
Source: Dyslexia. 2026 Apr 1;32(2):e70032. doi: 10.1002/dys.70032 (PMC13042268; doi:10.1002/dys.70032)
Supplement: Supplementary file 1 — Supporting Information: 1. [file DYS-32-e70032-s001.docx]

**Supplementary Data 1**

Table S1.1. *Bayesian independent samples t-tests (student) to compare the two groups on the baseline variables Socioeconomic status (SES), IQ, and Vocabulary*

|  | Dyslexia Only | |  | ADHD + Dyslexia | |  |  |  |
| --- | --- | --- | --- | --- | --- | --- | --- | --- |
| Variable | M | (SD) |  | M | (SD) |  | BF_10_ | Error % |
| SES ^a^ | 35519 | (4003) |  | 35007 | (6253) |  | 0.35 | 0.003 |
| IQ ^b^ | 103.81 | (10.70) |  | 97.51 | (7.82) |  | 1.30 | 0.005 |
| Vocabulary ^c^ | 99.38 | (6.32) |  | 100.38 | (12.31) |  | 0.35 | 0.003 |

*Note:*

n = 16 for all groups, except for SES in the ADHD+Dyslexia group (n = 15) due to one missing value.

**^a^ SES** is indexed by the median net household income of the neighborhood (based on postal code, as reported by the Dutch Bureau of Statistics (Van Leeuwen & Venema, 2025)) for each participant.

**^b^ IQ** is measured by the Dutch version of the Wechsler Intelligence Scale for Children.

**^c^ Vocabulary** is measured by the PPVT (Peabody Picture Vocabulary Test, standardized score).

Figure S1.1. *Raincloud plots of baseline variables SES, IQ, and Vocabulary (group 1 = Dyslexie only, group 2 = ADHD+Dyslexia)*

**Reference**

Van Leeuwen, N., & Venema, J. (2025). *Statistische gegevens per vierkant en postcode 2022-2021-2020-2019* [Statistical data per square and postal code 2022-2021-2020-2019]. Centraal Bureau voor de Statistiek (CBS). https://www.cbs.nl/nl-nl/longread/diversen/2023/statistische-gegevens-per-vierkant-en-postcode-2022-2021-2020-2019
