# Supplementary material for: Short Report: Intervention of Reading and Spelling Problems in Children With Co‐Occurring Attention‐Deficit Hyperactivity Disorder and Dyslexia
Source: Dyslexia. 2026 Apr 1;32(2):e70032. doi: 10.1002/dys.70032 (PMC13042268; doi:10.1002/dys.70032)
Supplement: Supplementary file 2 — Supporting Information: 2. [file DYS-32-e70032-s002.docx]

**Supplementary Data 2**

Bayesian repeated-measures analyses of variance with two factors, Group and Time. Factor Group has two levels (ADHD+Dyslexia vs. ADHD+Dyslexia+3^rd^ Diagnosis), and factor Time has two levels (pretest vs. posttest). Results do not support a main effect of Group nor an interaction-effect between Group and Time.

Table S2.1. *Model comparison for word reading fluency and spelling*

| Models | P(M) | P(M\|data) | BF_M_ | BF_10_ | Error % |
| --- | --- | --- | --- | --- | --- |
| (A) Reading Fluency 3DM |  |  |  |  |  |
| Time | 0.250 | 0.640 | 5.336 | 1.000 |  |
| Time + Group | 0.250 | 0.360 | 1.686 | 0.562 | 1.272 |
| Null model | 0.250 | 3.241×10^-5^ | 9.723×10^-5^ | 5.063×10^-5^ | 0.668 |
| Group | 0.250 | 1.485×10^-5^ | 4.455×10^-5^ | 2.320×10^-5^ | 1.552 |
|  |  |  |  |  |  |
| (B) Reading Fluency OMT |  |  |  |  |  |
| Time | 0.250 | 0.570 | 3.983 | 1.000 |  |
| Time + Group | 0.250 | 0.429 | 2.257 | 0.753 | 2.811 |
| Null model | 0.250 | 2.037×10^-4^ | 6.113×10^-4^ | 3.572×10^-4^ | 1.720 |
| Group | 0.250 | 1.234×10^-4^ | 3.702×10^-4^ | 2.164×10^-4^ | 1.851 |
|  |  |  |  |  |  |
| (C) Spelling |  |  |  |  |  |
| Time | 0.250 | 0.596 | 4.429 | 1.000 |  |
| Time + Group | 0.250 | 0.404 | 2.032 | 0.677 | 2.376 |
| Null model | 0.250 | 1.976×10^-5^ | 5.929×10^-5^ | 3.315×10^-5^ | 0.723 |
| Group | 0.250 | 1.024×10^-5^ | 3.073×10^-5^ | 1.718×10^-5^ | 1.491 |

*Note*. All models include subject, and random slopes for all repeated measures factors.

Figure S2.1. *Raincloud plots of pretest and posttest scores per group (ADHD+Dyslexia vs. ADHD+Dyslexia+3^rd^ Diagnosis)*

**
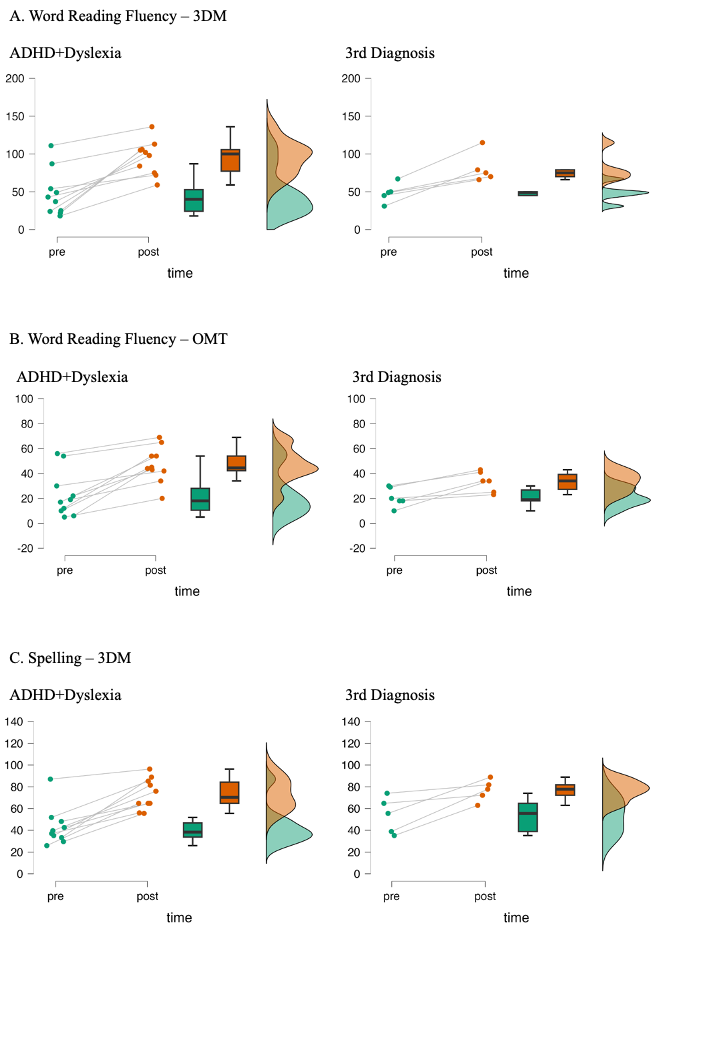
**
