# Supplementary material for: Short Report: Intervention of Reading and Spelling Problems in Children With Co‐Occurring Attention‐Deficit Hyperactivity Disorder and Dyslexia
Source: Dyslexia. 2026 Apr 1;32(2):e70032. doi: 10.1002/dys.70032 (PMC13042268; doi:10.1002/dys.70032)
Supplement: Supplementary file 3 — Supporting Information: 3. [file DYS-32-e70032-s003.docx]

**Supplementary Data 3**

Table S3.1. *Analysis of effects for word reading fluency and spelling*

| Effects | P(incl) | P(excl) | P(incl\|data) | P(excl\|data) | BF_incl_ |
| --- | --- | --- | --- | --- | --- |
| (A) Reading Fluency 3DM |  |  |  |  |  |
| Time | 0.600 | 0.400 | 1.000 | 1.864x10^-11^ | 3.576x10^10^ |
| Group | 0.600 | 0.400 | 0.900 | 0.100 | 6.022 |
| Time ✻ Group | 0.200 | 0.800 | 0.237 | 0.763 | 1.241 |
|  |  |  |  |  |  |
| (B) Reading Fluency OMT |  |  |  |  |  |
| Time | 0.600 | 0.400 | 1.000 | 8.549x10^-10^ | 7.799x10^8^ |
| Group | 0.600 | 0.400 | 0.778 | 0.222 | 2.337 |
| Time ✻ Group | 0.200 | 0.800 | 0.198 | 0.802 | 0.987 |
|  |  |  |  |  |  |
| (C) Spelling |  |  |  |  |  |
| Time | 0.600 | 0.400 | 1.000 | 1.124x10^-9^ | 5.932x10^8^ |
| Group | 0.600 | 0.400 | 0.992 | 0.008 | 82.654 |
| Time ✻ Group | 0.200 | 0.800 | 0.638 | 0.362 | 7.055 |
